# FEATURES OF LEARNING FROM HIGH-FREQUENCY COGNITIVE ASSESSMENTS AS DIGITAL BIOMARKERS OF COGNITIVE CHANGE

**DOI:** 10.1093/geroni/igad104.2043

**Published:** 2023-12-21

**Authors:** Zita Oravecz, Joachim Vandekerckhove, Cuiling Wang, Mindy Katz, Jonathan Hakun, Martin Sliwinski

**Affiliations:** Pennsylvania State University, State College, Pennsylvania, United States; Cognitive Sciences, Irvine, California, United States; Albert Einstein College of Medicine, Bronx, New York, United States; Albert Einstein College of Medicine, Bronx, New York, United States; Pennsylvania State University, State College, Pennsylvania, United States; Pennsylvania State University, State College, Pennsylvania, United States

## Abstract

In measurement burst designs, participants’ cognitive performance is measured multiple times per day, for several days, forming a measurement burst. Ideally, these are repeated once or twice a year as people age. Such rich longitudinal data are generated by multiple processes (e.g., aging and learning) that operate on multiple timescales. We propose a Bayesian process model that can extract person-specific, substantively meaningful features of learning and change from such data. We show how to model retest gains across measurement bursts, as well as warm-up effects within a burst, while quantifying change across bursts in peak performance and accounting for short-term within-person variability. Individual differences in these features are also linked with psychosocial variables and biomarkers of cognitive decline in a one-step analysis. We also highlight how this approach allows for drawing intuitive inferences on cognitive decline with Bayesian posterior probabilities.

